# The Legal Value of the Testimony of the Insane

**Published:** 1885-08-20

**Authors:** 


**The Legal Value of the Testimony of the Insane.**—The United States Supreme Court has decided that the testimony of "a lunatic or person affected with insanity is admissible as a witness if he has sufficient understanding to apprehend the obligation of an oath, and to be capable of giving a correct account of the matters which he has seen or heard with reference to the question at issue ; and whether he has that understanding is a question to be determined by the court, upon examination of the party himself and any competent witness who can speak to the nature and extent of his insanity.—*The College and Clinical Record.*

